# Typhus or Ship Fever

**Published:** 1847-12

**Authors:** 


10. *Typhus or Ship Fever.*—The following extract from observations by John H. Griscom, M. D., in the New York Hospital, which we take from the *N. Y. Journal of Medicine*, will be found interesting, as the disease of which the author speaks, is attracting much attention. E.

The *treatment* of this disease was based upon the idea of its proximate cause being *mainly* a vitiated, deficient and innutritious condition of the blood. I say *mainly*, because I have no particular theory as to the real nature of the disease, whether produced by a specific poison entering the system from without, as is maintained by some, or by a partial decomposition of the blood by others, or by a disorganization of the solids by a third party, etc. The most important point in my estimation to be considered, being its treatment, I have been disposed to look chiefly at its *remote causes*, and to endeavor to ascertain from a contemplation of them, what is required to overcome their effects.

The remote causes are two in number: 1st, an insufficiency of food, and 2d, the inhalation of a vitiated air. The first of these must necessarily produce an exhausted nutritive condition of the blood;—that fluid, under a protracted privation of nutriment, will not only be diminished in quantity, but its red globules, it is reasonable to suppose, will become deficient in number and in those properties which are believed necessary to the health of the organism. Both these consequences are aggravated and increased by the second cause; for in the atmosphere of the steerage of a passenger ship, crowded to the utmost limit of the law, there must necessarily, one may easily believe, be not only a deficiency of oxygen, but an actual presence of other gases, whose chemical action upon the blood cannot but be deleterious, depriving it still further of its healthy properties.

I may be told that this brief view of the causes and character of ship fever is insufficient to account for the *febrile symptoms*,—that there is nothing in starvation, or want of oxygen, or the presence of deleterious gases, to produce *fever*. If any one who should raise this objection to the insufficiency of my position will tell us *what fever is*, I might then be able to discover a connection between it and the causes I have named. Until the *specific* nature of fever is demonstrated, it is in vain to argue about the nature of its causes, or to endeavor to trace the modus operandi of the influences which are supposed to produce it. But if we are to understand by fever, the frequent pulse, hot skin, thirst, etc., etc.,—then I answer, that ship fever, as it has been presented to us this year, is in many instances not a fever at all. Repeatedly have we seen patients brought from on ship board without a single symptom of fever, with pulse below the natural standard, skin moist and cool, fauces not dry, no thirst, and yet the body covered with petechiæ, the eye congested, the senses benumbed, and most of the other symptoms of the *Typhus condition.*

Confining our attention to this simple view of the causes of Ship Fever, we find little else to do than to counteract their effects. The means for this are clearly indicated, and may be classed under three general heads.

1st. To maintain the continuity of the body, and sustain its nervous energy, by stimuli, until we are enabled,

2d. To improve the quantity and character of the blood by appropriate nourishment, and

3d. To oxygenize the blood thoroughly by pure air.

For the first indication, after giving a warm bath, (an invariable rule where it could be borne,) the most powerful and direct stimulants were found necessary. Brandy and carbonate of ammonia consiitute the main reliance; and during my attendance I have been astonished to observe what enormous quantities of these remedies will be borne in this disease. As an instance, I may mention the case of a girl about fifteen years of age, who took about 5 pints of brandy every day for 5 days, and for two weeks longer from 2 to 3 pints daily. At the same time she was taking 10 grains of carb. ammonia every 15 minutes, amounting to two ounces in twenty-four hours, besides soup and other nutriments. And all this without the least manifestation of excitement, or injury to the stomach or bowels, such was the intensity of the disease. She was under this treatment nearly three weeks, before any very decided symptoms of improvement were manifested; unfortunately, before time elapsed to observe the ultimate result in this case, and just as she was beginning to feel the good effects of the treatment, the patient had to be discharged "relieved," being removed from the Hospital by her parents. Many other cases might be cited, in which it was necessary to continue, night and day, to ply these remedies unceasingly;—a very short respite was frequently sufficient to put the patient back decidedly, and a vast number of the cures were undoubtedly due to the faithfulness with which these means were applied. Where the circulation was unusually languid, or the extremities were cold, sinapisms, and artificial warmth were very valuable.

To answer the second indication, the patients were fed at frequent intervals with nutritious soups, arrow root or gruel, with wine or brandy, milk punch, egg-nog, beef, chickens, etc. etc.

Upon the third indication, pure air, I may remark, that on several occasions the necessity for it was strongly marked. The pressure for admissions several times became so urgent, that the bounds of prudence were quite overstepped, as was indicated by the fact that in certain of the wards which were most crowded, and contained the worst cases, the recoveries became more protracted, and the relapses more frequent. It became necessary to close two of the wards in the north building, and to have them thoroughly cleansed and purified. After this operation, and on confining the number of patients in them to a reasonable limit, a decided improvement was manifested in the rapidity of recoveries and convalesence. The position of a patient's bed in a ward, was observed to have an influence over his treatment. In the corners of the rooms the patients got along more slowly than in the central parts, or near the doors or windows;—and I frequently found that when a patient had been lying for several days in a part of a ward most distant from the windows, and was not doing well, a removal of his bed right under a window, would, in twenty-four hours, produce a decided change in the symptoms for the better.

Although this was the general course of treatment, it was frequently varied to suit the condition of the patient. Occasionally a case would present a degree of excitement, with hot and dry skin and thirst, which called for the spirit

Mindereri, ice in the mouth and to the head, and the mildest diet; sometimes gastric irritation with nausea would demand a mild emetic, such as an infusion of euper, perfol. If the pain and heat of the head were marked, dry cups to the temples, or forehead, or blisters behind the ears, and application of ice, would generally be found sufficient. Pneumonic symptoms with cough frequently complicated the case; when these occurred, Stoke's expectorant, with dry cups, or vesication of the chest, formed the principal addition to the other treatment.

Sometimes there would occur such a combination of general prostration external heat and dryness, as to indicate a combined stimulant and febrifuge treatment; such, for example, as the administration of carb. ammon.; or a half ounce of brandy, alternately every hour or two hours, with a half ounce of spirit. Minder.; and so frequent and sudden were the changes, in many instances, from one condition to the other, an almost constant watching was necessary to withhold the one or the other, and again resume it. In fact, the varieties and shades of symptoms were almost infinite, and called for an endless variation in the means of relief. To enumerate them would take more time and space than could be reasonably asked. There were many cases, however, for which no other treatment was necessary than good diet and cold water. Cleanliness, pure air, and food, appeared all-sufficient for the removal of the disease, even in well-marked cases, not a particle of medicine being administered to them.

*Local Complaints.*—A tendency to Erysipelas prevailed in some of the wards during the whole of my attendance. No exact account has been rendered of the number of Typhus cases in which it occurred, but they probably amounted to about 20. It attacked the face and head in every instance. Its treatment did not vary from that required under ordinary circumstances, except so far as the Typhoid condition of the patient required. Cooling applications to the part affected, with internal stimulation, constituted the general course, with complete success in every instance but one, who died from the Typhus fever, his condition from the first having been desperate. One case will justify more detailed notice. It was that of a nurse, who contracted the fever from his patients; he recovered, and returned to duty; in two weeks he had a relapse, from which he recovered slowly, and soon after getting to duty again, had a second relapse, accompanied with Erysipelas in the face and head. The frequent attacks of the fever had so reduced him, that now, with the Erysipelatous complication, it seemed almost in vain to prescribe for him, especially, when in a few days the most serious symptoms of all occurred, viz: great heat of head and evident congestion of the brain, attended with delirium and total unconsciousness. His eyes were entirely closed by the swelling of the integuments, the tongue was dry and black, the pulse frequent and feeble. A more hopeless combination of circumstances could hardly be imagined. Depletion, even local, from the head, was totally forbidden by the prostration; and stimulation by brandy, and carb. ammon., while contributing to sustain the general strength, seemed to add to the difficulty in the head. In this posture I resolved upon the exhibition of a remedy, which I am not aware has been before tried under similar circumstances. He was directed to have *five grains of Hydriodate of Potassa every hour.* The effect of this (supposing the "post hoc" to be the "propter hoc") was magical, for in 24 hours there was a decided diminution of the cerebral congestion, and the patient rapidly recovered. The remembrance of the efficacy of this remedy as a deobstruent in certain conditions of the brain in children, especially in hydrocephalus, both the true and stimulated form, induced the hope of obtaining a similar usefulness from it in the case I have mentioned. Further observations, it is hoped, will test its efficacy in the same condition of the adult brain.

*Dysentery* was also a frequent complication of the fever, and a majority of the deaths which occurred in the latter part of my service, were produced by it. The force of the disorder fell so frequently upon the intestinal canal, as to assume an endemic character in the month of September; and the occurrence of this complication, accompanied as it generally was by purulent bloody stools, was justly dreaded.

Some of the cases which, at the outset, threatened most seriously, yielded to treatment; but it would seem, judging from the post-mortem appearances, and the rapidity with which the patients sank, that the disorganization must, in many cases, have proceeded to an incurable degree before our attention was called to it by the patient. This was chiefly owing to the anomalous fact, that, even in some of the severest forms of the dysentery, there was little or no pain evinced.

The degree of disorganization of the mucous coat, in those cases which were post mortemed, was most extraordinary, surpassing, in many instances, anything before within my experience. In some cases not a trace of healthy mucous membrane was discernible in the large intestine, from the ilio-cœcal valve to the anus; but from every point blood, or mucus, or pus, appeared to have been discharged, clearly demonstrating the utter incurability of the disease. Copious deposits of lymph-like matter were seen, forming *bas-relief* patches, and giving the idea, without close observation, of ulceration at the intermediate points. In one particularly severe case, the entire mucous coat of the colon bore a resemblance to the rough bark of a tree, the elevations of the new deposits were so great and uneven. The color in this case was greenish.

When this complication was diagnozed sufficiently early, the administration of Tannin, and nit. argent., both by mouth and enemata, opiates, blisters, and bland diet, frequently diminished the force of the disease, and sometimes with a successful issue; but the impossibility of direct applications to the ulcerated surfaces, and the necessity of dependence on general treatment for local disease, placed this disorder amongst the most dreaded.

When *Diarrhœa* formed the principal complication, the great sheet anchor was opium. This rarely failed to keep it in check, if administered to a sufficient extent. It was sometimes prescribed in conjunction with tannin, one to three grains of the latter, from three to twelve times a day, or with nitrate of silver, $\frac{1}{4}$ to $\frac{1}{2}$ a grain every 2, 4, or 6 hours, according to the severity of the symptom: but generally opium alone was found sufficient, while it seemed to have a happy effect, also, upon the febrile symptoms. Held in abeyance by the opium, the diarrhœa, as the general disease abated, and the strength improved, would gradually disappear.

One case of typhus was complicated with pregnancy of about four months. The patient aborted, but the uterus had not sufficient power to expel the secundines. No hemorrhage ensuing, and the patient being too much prostrated to endure any direct treatment for their removal, they were left, and were retained five days, when they were spontaneously expelled, and the patient recovered in the ordinary time.

*Delirium*, which was a frequent complication, was usually controlled by cups or blisters to the temporal and mastoid regions, and by the exhibition of morphine or Dover's powder. Large doses of these sedatives were borne with entire impunity, and the most happy effects. In two cases, (young females) the mind continued to be disturbed so long after recovery, as to give rise to apprehensions of confirmed insanity, but they happily recovered perfectly.

There were several other complications, such as jaundice, laryngitis, &c., but being only isolated cases, require no special notice.

The following tables prepared from the books, exhibit the results of the treatment of typhus:—

| | |
|---|---|
| Cases remaining June 30th - - - | 134 |
| Admitted in July - - - - - | 100 |
| " August, - - - - | 126 |
| " September, - - - - | 107 |
| Whole number cases of typhus in three months, | 467 |

Of these there were discharged,

| | Cured. | Relieved. | Disorderly. | Request. | Died. |
|---|---|---|---|---|---|
| In July, | 128 | 1 | 1 | 2 | 14 |
| In August, | 108 | | | 3 | 13 |
| In September, | 94 | | | 1 | 19 |
| | 330 | 1 | 1 | 6 | 46 |

| | |
|---|---|
| Whole No. cases typhus discharged, | 384 |
| " " " remaining Oct. 1st. | 83 |
| Per centage of cures, | 85.93 |
| " " relief | 2.10 |
| " " deaths | 11.97 |

In a considerable number of instances, however, in which the result was fatal, death was attributable not directly to typhus fever, but to some other disease, which made its onset in the enfeebled state of the patient during convalescence; so that the ratio of deaths from typhus alone would be materially less, amounting, perhaps, to 8 or 9 per cent.

Especially was this the case, as already stated, in September, when dysentery prevailed endemically, and was the immediate cause of death in many cases of typhus, but in which the fatal result is assigned to the credit of the latter disease, on the books. One of the deaths recorded as by typhus, entered the Hospital with a *fifth attack of delirium tremens*, from which he recovered; but before being discharged, being subjected to the prevailing infection, he gradually sank, though with hardly sufficient character of typhus to be added to the list. There are probably very few persons capable of surviving five severe attacks of mania à potu.

| | | |
|---|---|---|
| The whole number of *Autopsies* in the three months (of typhus) was | | 10 |
| The head was the principal part affected in | 1 | |
| The chest | 1 | |
| The head and abdomen both | 6 | |
| Nothing special was found in | 2 | |
| | — | 10 |

| | |
|---|---|
| Peyer's plates were prominent in | 4 |
| " " ulcerated in | 2 |
| | — |
| | 6 |

In every case where the bowels were affected in either of the ways specified, the membranes of the brain were congested, or there was free serous effusion, or both.

In one case in which the plates of Peyer were prominent, they were enormously large—some of them near the end of the ilium, exceeding 6 inches in length.

There were two other autopsies of persons who had been entered as cases of Feb. Remit., but the description of which leaves room to doubt whether they had not the typhus infection. In one, the membranes of the brain were congested, and there was bloody effusion. In the other there was effusion in the cranium, and the liver was enlarged, congested, and bronze.

